# Diversity and Adaptations of *Escherichia coli* Strains: Exploring the Intestinal Community in Crohn’s Disease Patients and Healthy Individuals

**DOI:** 10.3390/microorganisms9061299

**Published:** 2021-06-15

**Authors:** Maria N. Siniagina, Maria I. Markelova, Eugenia A. Boulygina, Alexander V. Laikov, Dilyara R. Khusnutdinova, Sayar R. Abdulkhakov, Natalia A. Danilova, Alfiya H. Odintsova, Rustam A. Abdulkhakov, Tatyana V. Grigoryeva

**Affiliations:** 1 Institute of Fundamental Medicine and Biology, Kazan Federal University, 420008 Kazan, Russia; MIMarkelova@kpfu.ru (M.I.M.); boulygina@gmail.com (E.A.B.); AVLajkov@kpfu.ru (A.V.L.); dilyahusn@gmail.com (D.R.K.); SRAbdulhakov@kpfu.ru (S.R.A.); 1Tatyana.Grigoreva@kpfu.ru (T.V.G.); 2 Department of Outpatient Therapy and General Medical Practice, Kazan State Medical University, 420012 Kazan, Russia; 3 Department of Gastroenterology, Republican Clinical Hospital of Tatarstan Republic, 420064 Kazan, Russia; danilova.natalya.87@mail.ru (N.A.D.); alsuabdulkhakova@yandex.ru (A.H.O.); 4 Department of Hospital Medicine, Kazan State Medical University, 420012 Kazan, Russia; rustemabdul@mail.ru

**Keywords:** Crohn’s disease, *Escherichia coli*, whole-genome sequencing, shotgun metagenome sequencing

## Abstract

Crohn’s disease (CD) is characterized by a chronic, progressive inflammation across the gastrointestinal tract with a series of exacerbations and remissions. A significant factor in the CD pathogenesis is an imbalance in gut microbiota composition, particularly the prevalence of *Escherichia coli*. In the present study, the genomes of sixty-three *E. coli* strains from the gut of patients with CD and healthy subjects were sequenced. In addition, eighteen *E. coli*-like metagenome-assembled genomes (MAGs) were reconstructed from the shotgun-metagenome sequencing data of fecal samples. The comparative analysis revealed the similarity of *E. coli* genomes regardless of the origin of the strain. The strains exhibited similar genetic patterns of virulence, antibiotic resistance, and bacteriocin-producing systems. The study showed antagonistic activity of *E. coli* strains and the metabolic features needed for their successful competition in the human gut environment. These observations suggest complex bacterial interactions within the gut which may affect the host and cause intestinal damage.

## 1. Introduction

*Escherichia coli* is a very important facultative aerobic inhabitant of the human intestine and part of the normal flora. *E. coli* is among the first bacterial species to colonize the intestine during infancy, forming the human gut microbiota [1]. Imbalances in host–commensal interaction may lead to intestinal and extraintestinal infections [2]. Many works have been devoted to metagenomic investigations, discovering the changes in microbiota which promote intestinal inflammation [3,4,5,6,7,8]. Thus, a decrease in biodiversity, accompanied by an increased abundance of *E. coli*, was found to be associated with CD [9,10,11]. However, a single *E. coli* strain specific to CD has not been discovered to date.

Closely related microorganisms, especially at the strain level, occupy similar ecological niches, leading to an intense competition which can promote rapid phenotypic evolution [12]. Serotyping [13,14,15,16,17], multilocus sequence typing (MLST) [18,19], and phylotyping by Clermont’s method [20] are widely used to study the strain-level variability in *E. coli*. A hypothesis of the potential for O-antigens of intestinal *E. coli* to induce immune stimulation and play a role in the pathogenicity of IBD was formed in the 1970s. However, associations of specific O-serotypes with the disease were not found [21]. At present, conventional serotyping is being replaced by more rapid and accurate molecular typing [22,23]. High-throughput sequencing significantly expanded opportunities in genetic diversity studies into *E. coli*. Using the WGS technique, a number of *E. coli* genomes from CD patients have been characterized; however, most studies have focused on pathogenic clinical isolates [24,25,26,27,28,29]. Attempts to explain the role of *E. coli* in CD pathogenesis have drawn attention to adherent-invasive strains (AIEC). Research into CD has revealed the ability of *E. coli* to adhere to intestinal epithelial cells and survive within macrophages, which allows bacteria to move across the human intestinal barrier and potentially induce the formation of granulomas [30,31,32,33]. However, the precise mechanism of CD pathogenesis remains unclear. Studies that focus on mucosa-associated *E. coli* often overlook fecal commensal strains, which play an important role in the microbiota. It has been shown that the probiotic strain Nissle 1917 affects intestinal permeability and improves barrier integrity in IBD patients [34,35]. The strain outcompetes enteropathogens and reduces colitis [36,37]. However, in healthy people, a well-established microbiota prevents the integration of the probiotic strain into the existing community [38]. The replacement of pro-inflammatory *E. coli* with competitive strains of healthy subjects might be beneficial in reducing inflammatory activity. As another step towards a better understanding of the role of *E. coli* in a healthy and inflamed intestinal community, a cohort study was performed using different physiological and molecular approaches. To explore the relationships between strains within a healthy and inflamed intestine community, we isolated and thoroughly characterized fecal *E. coli,* and assessed their competitive potential and metabolic features.

## 2. Materials and Methods

### 2.1. E. coli Collection and Phylotyping

A total of 82 human fecal samples (40 CD patients and 42 healthy volunteers) were collected at the Kazan Federal University Hospital (Kazan, Russian) and stored at −80 °C until needed. Patients with CD were diagnosed by clinical and endoscopic examination with further histological confirmation. A complete list of inclusion/exclusion criteria for CD patients and healthy individuals has been described previously [39]. Individuals taking antibiotics six months before sample collection were excluded from the study. For shotgun metagenomic analysis, all samples were sequenced using the Illumina NextSeq 500 platform and fully characterized in our previous work [39]. The fecal samples were prepared according to the described previously protocol [40]. Briefly, the stool samples (0.1 g) were resuspended in PBS and inoculated on a selective medium (Endo agar). Up to 10 colonies from each plate were selected for species identification using matrix-assisted laser desorption/ionization (MALDI) Biotyper System (Bruker, Bremen, Germany). For DNA isolation with PureLink Genomic DNA Mini kit (Invitrogen, Thermo Scientific, Carlsbad, CA, USA), each *E. coli* colony was grown in the Luria-Bertani broth (LB) at 37 °C with shaking overnight. Further typing was performed through quadruplex PCR according to Clermont’s method [20], followed by in silico validation.

### 2.2. Whole-Genome Sequencing and Analysis

In total, of 521 cultivated colonies from 32 individuals (14 CD patients and 18 healthy controls), 97 distinct *E. coli* isolates were identified, then sequenced by the Illumina MiSeq platform provided by Interdisciplinary Centre for Shared Use of Kazan Federal University, and analyzed as described previously [40].

In addition, metagenome-assembled genomes (MAGs) from all 82 human fecal samples were reconstructed with metaSPAdes v. 3.14.1 [41]. Contigs longer than 1000 nucleotides were binned with Maxbin v. 2.2.7 [42]. Only 18 high-quality MAGs with a completeness of more than 80% and a contamination rate of less than 5%, as estimated by CheckM v. 1.0.11 [43], were considered for further analysis.

A phylogenetic tree was constructed using PhyloPhlAn 3.0 [44] and visualized with iTOL (https://itol.embl.de, accessed on 8 February 2021) [45]. Multidimensional scaling (MDS) plots were built on the whole-genome average nucleotide identity (ANI) distances computed with FastANI v 1.32 [46].

Serotypes were assigned using the SerotypeFinder-2.0 tool [22]. All known Clermont’s phylotypes were in silico assigned using the EzClermont tool [47] Multi-Locus Sequence Typing (MLST) was performed with MLST 2.0 [48] based on two main methods: Achtman’s scheme with internal fragments of seven housekeeping genes (*adk*, *fumC*, *gyrB*, *icd*, *mdh*, *purA*, *recA*) [49] and a Pasteur scheme consisting of eight genes (*dinB*, *icdA*, *pabB*, *polB*, *putP*, *trpA*, *trpB*, *uidA*) [50].

Nucleotide sequences were aligned to the custom virulence genes database using BLASTn (with a cut-off of 80% identity over an 80% virulence gene length, E < 10^−16^). Differences between the frequency of occurrence of virulence genes were calculated using Fisher’s exact test with Benjamini–Hochberg correction for multiple comparisons.

### 2.3. In Vitro Competition Assay

The halo assay was performed according to Ohno [51] with some modifications. The tester *E. coli* K-12 substr. MG1655 was inoculated into the 0.8% agar and poured onto a bottom 2% LB agar plates. A series of 10-fold dilutions of each competitor strain (starting with OD600 = 1.0) were spotted (3 μL) on the tester strain and incubated at 37 °C overnight. Each experiment was repeated twice. Zones of at least 1 mm width were considered inhibition zones.

### 2.4. Antimicrobial Resistance

All isolates were tested for antimicrobial susceptibility with fluoroquinolones (ciprofloxacin, levofloxacin, moxifloxacin, nalidixic acid), aminoglycosides (amikacin), carbapenems (meropenem), monobactams (aztreonam), penicillins (ampicillin), polymyxins (polymyxin B), sulfanilamides (sulfanilamide), tetracyclines (tetracycline), and cephalosporins (cefazolin, cefepime, cefotaxime, cefuroxime) using a disc-diffusion test (DDT) according to EUCAST guidelines (https://www.eucast.org/ast_of_bacteria/disk_diffusion_methodology/, accessed on 3 June 2019). The *E. coli* strain ATCC 25922 was used as a control. Antibiotic resistance genes in isolated strains and MAGs were predicted using the Resistance Gene Identifier (RGI) and the CARD database [52]. Differences between the frequency of occurrence of resistance genes were calculated using Fisher’s exact test with Benjamini–Hochberg correction for multiple comparisons.

### 2.5. Biochemical Assay

Biochemical characterization of *E. coli* isolates was performed using ENTEROtest 24N (Erba Lachema, Brno, Czech Republic) according to the manufacturer’s recommendations.

## 3. Results

### 3.1. Phylogenetic Analysis of E. coli Strains Isolated from CD Patients and Healthy Subjects

In the current study, we screened 82 fecal samples of 40 CD patients and 42 healthy individuals collected at the Kazan Federal University Hospital (Kazan, Russia), for which shotgun sequencing has been performed (Table 1). According to the published metagenomic data [39], a higher proportion of *E. coli* was found in stool samples of CD patients compared to controls. From these stool samples, *E. coli* strains were carefully selected and identified. Of 32 samples (14 from CD patients and 18 from healthy individuals), 97 isolates were sequenced, followed by comparative genome analysis, which revealed 33 duplicates, i.e., isolates sequenced more than once due to varying colony size and morphology. Thus, 64 strains had unique genomes, including 26 isolates from CD patients, 37 isolates from the control group, and one contaminated genome, which was filtered out. Patients with cultivated *E. coli* were separated into three subgroups according to disease location: ileal (*n* = 3), ileocolonic (*n* = 4), and colonic CD (*n* = 7).

To comprehensively characterize fecal *E. coli* diversity, 18 high-quality *E. coli*-like MAGs (11 from CD patients and 7 from healthy controls) were reconstructed from the shotgun metagenome sequencing data of fecal samples. Four out of 18 MAGs were highly similar to the cultivated isolates from the same fecal samples, six were reconstructed from samples that failed to cultivate *E. coli*, and the rest were not isolated by cultivation but found in the metagenome data.

**Figure 1:**
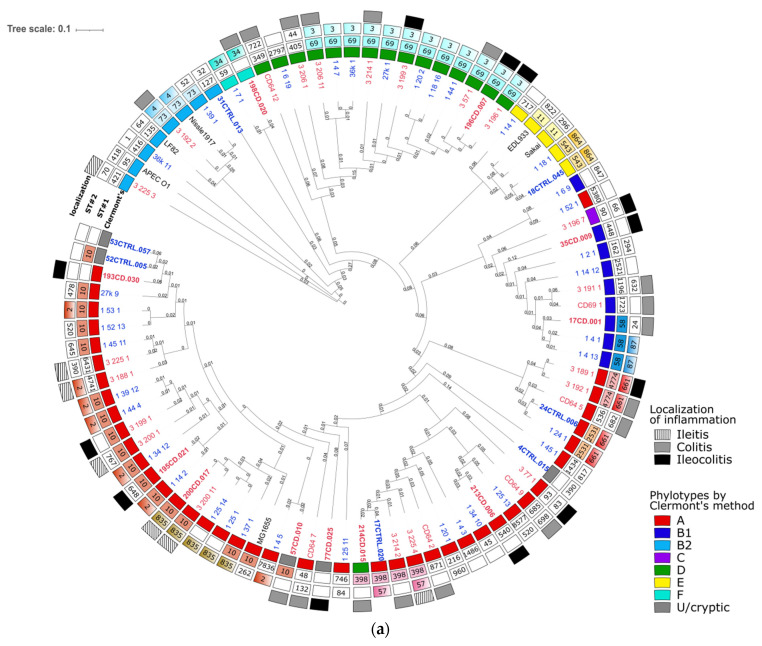

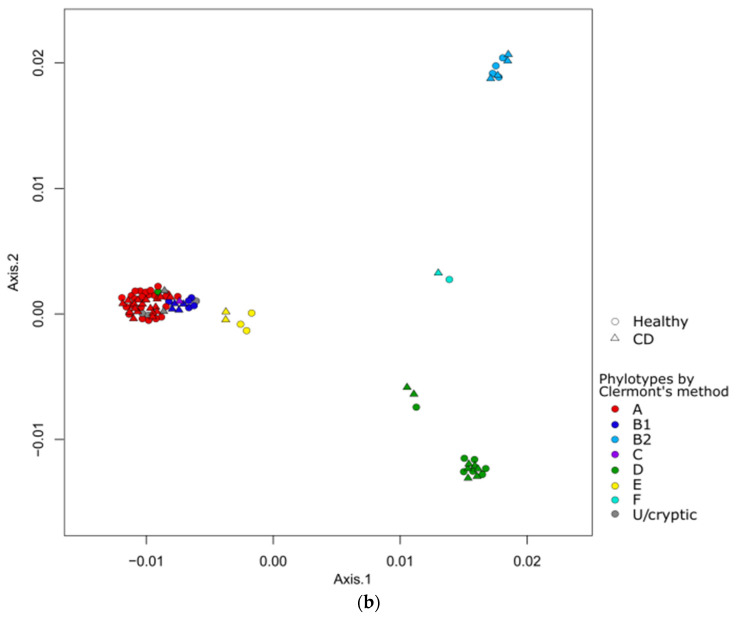
Comparative genome analysis of fecal *E. coli* strains isolated from patients with Crohn’s disease (CD) and healthy individuals. (**a**) Phylogenetic tree of 63 *E. coli* genomes and 18 MAGs (marked in bold) from healthy individuals (blue) and CD patients (red). Complete genomes of *E. coli* strains APEC O1, LF82, Sakai, EDL933, Nissle 1917, and K-12 substr. MG1655 were used as reference sequences (black). Phylogenetic groups by Clermont’s method [20] are highlighted on the inner ring. Sequence types (ST) by the Achtman’s scheme [49] and the Pasteur’s scheme [50] are marked as ST#1 and ST#2, respectively. Repeating STs are colored; (**b**) Multidimensional scaling (MDS) on average nucleotide identity (ANI) distance of all genomes is colored according to Clermont’s phylogroups.

The phylogenetic analysis was performed for the full set of the cultivated 63 *E. coli* genomes and 18 reconstructed MAGs using reference pathogenic (enterohemorrhagic EDL933 and Sakai, APEC O1, AIEC LF82) and non-pathogenic (Nissle 1917 and K-12 substr. MG1655) strains (Figure 1, Supplementary Materials Table S1). The phylogenetic tree showed a high similarity in analyzed genomes, and disease-associated clustering of *E. coli* was not observed, but strains were divided according to known phylogenetic groups [19]. The phylogroups A (49%) and D (20%) represented the majority of the strains, and the rest (B1, B2, C, E, and F) were less common. Based on phylogenetic typing, it was demonstrated that strains from patients with CD were indistinguishable from those isolated from healthy volunteers.

In addition to phylogenetic typing, we performed MLST analysis. According to Achtman’s MLST scheme [49], 63 cultivated isolates were distributed into 36 sequence types (ST#1 in Figure 1). The most common STs were ST10 (*n* = 12) and ST69 (*n* = 11), belonging to groups A and D, respectively. Four STs were identified more than once (ST58, ST73, ST398, ST4774). Reconstructed genomes expanded this list, with three STs found (ST127, ST448, ST540). The Pasteur scheme [50] divided *E. coli* strains into 30 sequence types (ST#2 in Figure 1). Nine STs occurred twice or more and comprised 59% of all strains (ST2, ST3, ST34, ST5, ST87, ST390, ST520, ST661, ST835). The most frequent types among strains were ST2 (*n* = 9) and ST3 (*n* = 11) assigned to A and D groups, respectively. Two STs were identified only in MAGs (ST24, ST32). Together, in accordance with both MLST schemes, two main clusters of closely related genotypes (ST69/ST3 and ST10/ST2) from different hosts were formed.

### 3.2. Genetic Features of E. coli Strains of CD Patients and Healthy Individuals

It is known that intestinal commensals interact closely with the human immune system [53], and their resistance to host defense responses is partially dependent on surface structures, particularly O-antigens [54,55]. Therefore, we performed in silico serotyping, which revealed a pool of serologically distinct *E. coli* strains circulating in the gut community. Among the sequenced genomes and MAGs, 92% (34/37) and 86% (38/44) of *E. coli* were O- and H-typable in patients with CD and healthy controls, respectively. Twenty-seven different serotypes in patients with CD, and 32 in the healthy group, were discovered in total. Unrelated individuals from both cohorts shared five common serotypes, including O17/O44:H18 (*n* = 5), O144:H45 (*n* = 3), O6:H1 (*n* = 2), O25:H18 (*n* = 2), and O1:H7 (*n* = 2). Serotypes O8:H30 and O15:H18 occurred several times in healthy individuals, while O19:H4 and O128ac:H12 were found twice in patients with CD. The other strains displayed non-repeated combinations of O- and H-antigens. The most frequent O-antigen was O8 (8/81). The spectrum of antigens circulating in the human intestinal microbiota was discovered to be quite diverse; however, no association was found between strains of a specific serotype and CD (Supplementary Materials Table S1).

In order to assess virulence potential, the strains were screened for the presence of previously reported virulence genes [27,56,57], including genes encoding:

- Hemolysins (*hlyA*, *hlyF*);
- Toxins (*astA*, *sat*, *vat*, *pic*, *cnf1*, *cvaB*, *cvaC*, *clbB*);
- Adhesion factors (*iha*, *papC*, *papGIII*, *papGII*, *sfaS*, *focG*, *afaC*, *nfaE*, *fimH*, *fimC*, *bmaE*, *csgA*, *gafD*, *mat*, *tia*, *lpfA*, *lpfB*, *lpfC*, *tsh*, *ompA*), capsule antigens (*kpsM*, *kpsT*, *neuC*);
- Lipopolysaccharide biosynthesis proteins (*waaL*, *waaV*, *waaW*);
- Flagellar antigen (*fliC*) and flagellar regulators (*flhC*, *flhD*, *fliA*);
- Invasion factors (*ibeA*, *gimB*, *malX*);
- Peyer’s patch-specific virulence factor (*gipA*);
- Serum survival factors (*iss*, *trat*, *nlpI*);
- CRISPR-associated proteins (*cas1*, *cas6*, *cas_csy2*, *cas_csy3*);
- Porins (*ompC*);
- Defensin resistance proteins (*arlC*, *arlA*);
- *Yersinia* high-pathogenicity island (*fyuA*, *ybtA*, *ybtP*, *ybtQ*, *ybtT*);
- Iron uptake systems (*sitA*, *sitB*, *sitC*, *sitD*, *iucD*, *iutA*, *iroN*, *chuA*, *ireA*, *eitA*, *eitC*, *etsB*, *etsC*, *HRA-2*);
- Two-component regulatory system EnvZ/OmpR (*envZ*, *ompR*);
- Stress response proteins (*hfq*, *htrA*, *impK*, *rpoE*, *rpoS*, *dsbA*);
- Propanediol utilization enzyme (*pduC*);
- Type VI secretion systems (T6SSs).

**Figure 2:**
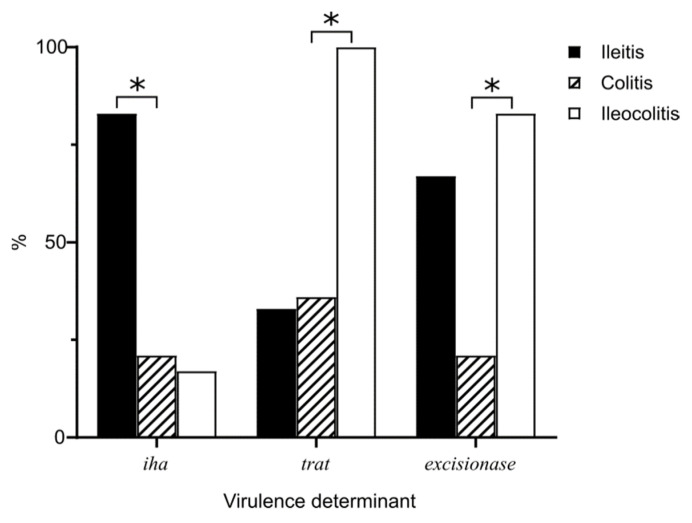
Frequency of occurrence of significantly different virulence genes in *E. coli* strains from patients with Crohn’s disease, depending on disease location (* *p* < 0.05, Fisher’s exact test).

However, the comparative analysis did not reveal significant differences in the distribution of virulence genes between isolates from healthy and affected groups (Supplementary Materials Table S2). Considering the disease location, a high frequency of *iha* gene (bifunctional enterobactin receptor/adhesin protein) was found in strains from patients with ileitis (Fisher’s exact test, p-value adjusted with Benjamin–Hochberg correction, *p* ≤ 0.05) compared to colitis and ileocolitis, while the serum survival factor TraT and excisionase (*p* ≤ 0.05) were more frequent in patients with colitis (Figure 2). Interestingly, the most common sequence types for *E. coli* strains (ST10, ST69) significantly differ from other STs regarding the number of virulence genes for adhesion factors, lipopolysaccharide biosynthesis proteins, iron uptake systems, type VI secretion system, *Yersinia* high-pathogenicity island, capsule antigens (*kpsT*), serum survival factor (*trat*), stress response protein ImpK, and propanediol utilization enzyme (*pduC*) (Fisher’s exact test, *p* < 0.05) (Supplementary Materials Table S3). Therefore, it was supposed that the repertoire of virulence determinants may not reflect the ability of the strains to cause the disease, but may contribute to successful competition in the bacterial community. T6SSs, in particular, are known to play a role not only in bacterial adherence and invasion, biofilm formation and motility [58], but in interbacterial competition as well [59]. In this study, T6SS-2 gene cluster was identified in all *E. coli* genomes typed to ST3 (ST69) and belonging to phylogroup D. In one healthy- and one-CD-derived strains of phylogroup B2, both T6SS-1 and T6SS-2 systems were found. T6SS-3 locus was revealed in only one strain from a healthy individual.

**Figure 3:**
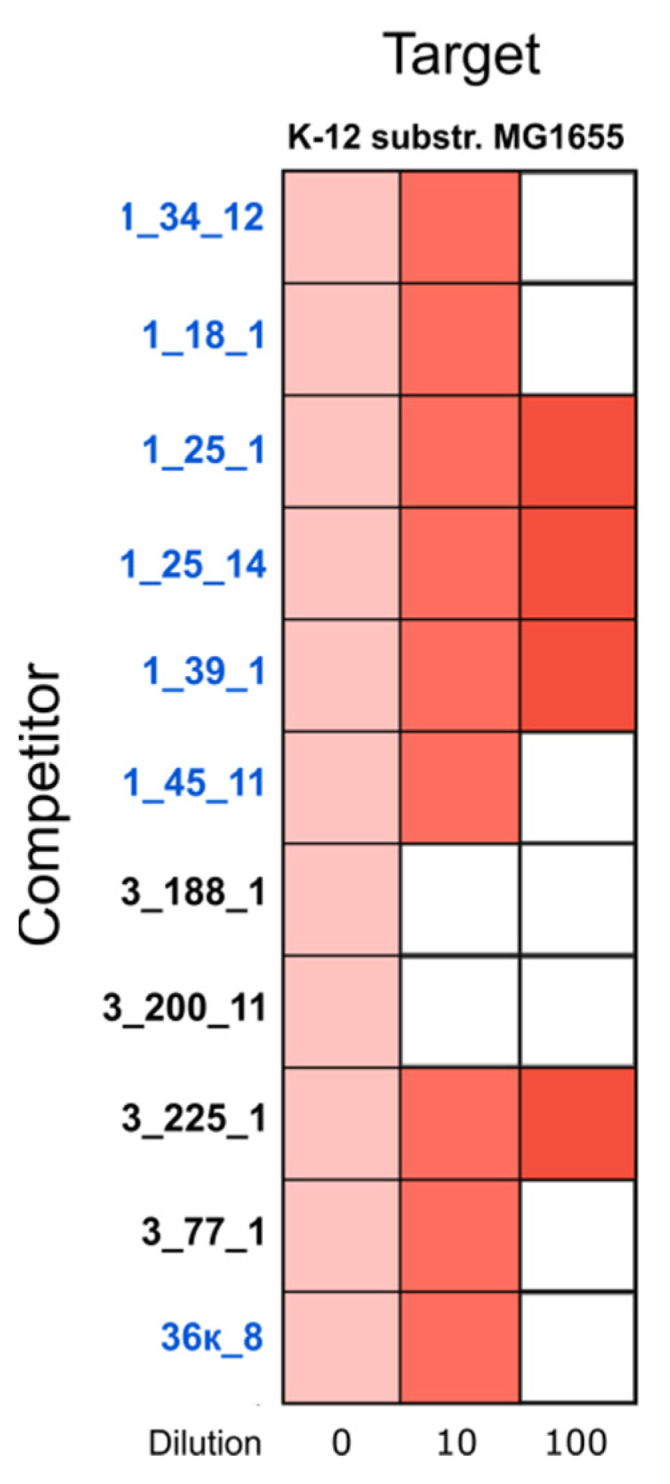
The antagonistic activity of *E. coli* strains from the gut against the strain K-12 substr. MG1655. Strains of healthy individuals are marked in blue. Red color indicates halo zone formation, white color—no halo. Deeper red corresponds to the lower concentration of the competitor strain.

Bacteriocin-producing systems benefit bacteria through their growth in a competitive environment as well. Screening of sequenced *E. coli* genomes revealed 28 strains (16 healthy and 12 CD-derived) containing genes for colicin and microcin production (Supplementary Materials Table S4). Microcin production systems, known for their promotion of gut colonization [60,61], were found equally in healthy and CD-derived strains. In isolated strains, the following genetic systems were identified for microcins of different classes: class I (MccC7, MccB17, MccJ25) [62], class IIa (MccV), and class IIb (MccH47, MccI47, MccM) [63]. Genes for the production of colicins (A, B, Ia, Ib, K, M, E1) were found in 23 strains (13 healthy and 10-CD derived). These isolates have the potential to acquire dominance under certain conditions. Indeed, according to CFU-counting data [40], all these strains dominated over other *E. coli* in the hosts. Therefore, we performed halo assays to determine the ability of the isolates to reveal antagonistic activity against other *E. coli* by overlaying the competitors on target bacteria K-12 substr. MG1655 (Figure 3). Only 11 isolates (four CD-derived and seven from healthy individuals) showed a phenotypic ability to inhibit the growth of the target strain.

### 3.3. Antibiotic Resistance and Its Genetic Determinants

**Figure 4:**
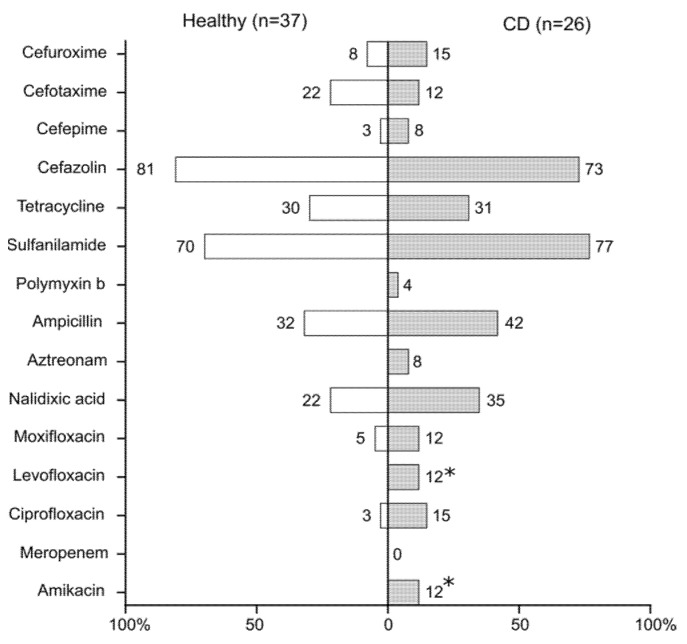
Susceptibility of *E. coli* strains from patients with Crohn’s disease (CD) and healthy individuals to the antibiotics. Numbers indicate the percent of non-susceptible strains (* *p* < 0.1, Fisher’s exact test).

Based on the disc-diffusion test, the majority of *E. coli* strains from CD (23/26) and healthy individuals (36/37) were non-susceptible to at least one of the tested antimicrobial drugs (Figure 4). All strains from both groups were sensitive to meropenem (carbapenems). The differences between patients with CD and healthy individuals were found in the susceptibility of the strains to amikacin and levofloxacin (Fisher’s exact test, *p* < 0.1), as well as to aztreonam and polymyxin (Supplementary Materials Table S5). Strains from both cohorts demonstrated non-susceptibility to 14/15 antibiotics tested, regardless of the location of intestinal inflammation. However, in one patient with ileal inflammation, both isolated strains were susceptible to all tested antibiotics. The strains that were non-susceptible to at least one agent in three or more antimicrobial categories and could be categorized as multidrug-resistant (MDR) strains [64] accounted for 14/26 (54%) of CD patients and 16/37 (43%) of individuals in the control group. Two patients with colonic inflammation harbored MDR strains which were non-susceptible to antibiotics of six different categories. The number of strains non-sensitive to ciprofloxacin, widely used for patients with active Crohn’s disease [65,66], was higher in the CD group, specifically: 4/26 (15%) in patients compared to 1/37 (3%) in controls.

Resistance genes predicted in silico were consistent with the phenotypic resistance revealed by the disk diffusion method (Supplementary Materials Table S6). All which were non-sensitive to tetracycline *E. coli* strains contained tetracycline efflux pump (*tetA*, *tetB*, *tetD*, *tetR*) genes. The inefficiency of fluoroquinolones could be due to plasmid-associated genes *qnrS1* and *qnrB19* and mutations in chromosome genes *gyrA* (S83L, D87N) and *parC* (S80I). The genes for class A beta-lactamases (TEM-1, TEM-150) and class D beta-lactamase OXA-1 were found in bacteria that were non-sensitive to ampicillin. The beta-lactamase TEM-150 only occurred in strains from healthy donors, not in CD patients. Although several strains carried aminoglycoside transferase genes, only one strain was detected to be non-sensitive to amikacin. Almost all *E. coli* strains harbored genes for AmpC type β-lactamases, which hydrolyze broad, extended-spectrum cephalosporins, genes of multidrug efflux systems MdtEF-TolC, EmrAB-TolC, EmrKY-TolC and AcrAB-TolC, MdtG and AcrD efflux pumps, and their regulators. To resist the antimicrobial activity of polymyxin, *E. coli* strains had genes that encode PmrF and PmrC enzymes modifying the antibiotic targets. However, only one strain from the CD patient was non-susceptible to polymyxin.

### 3.4. Metabolic Profiling of E. coli Strains

**Figure 5:**
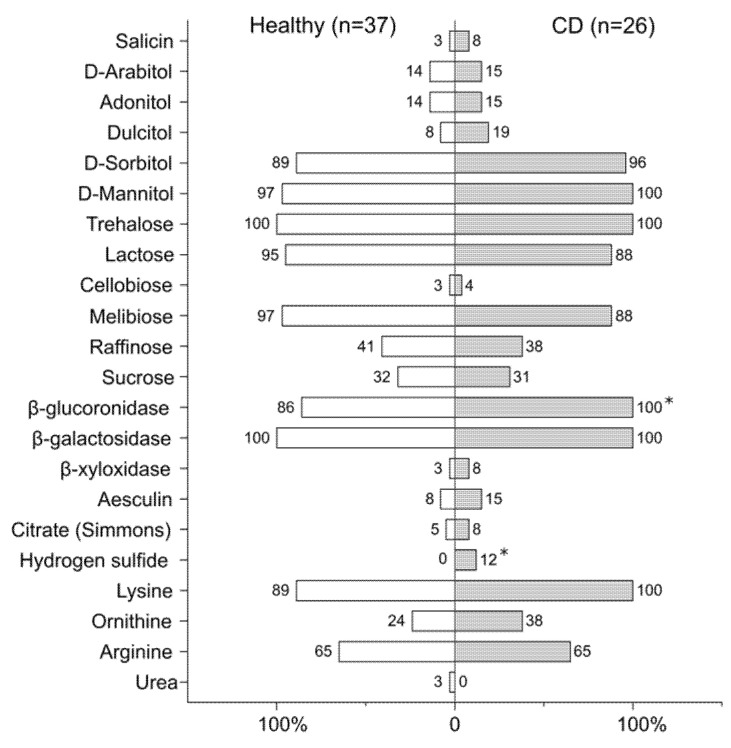
Distribution of biochemical features of *E. coli* strains from patients with Crohn’s disease and healthy individuals. Numbers indicate non-susceptible strains capable of utilizing corresponding carbohydrates (* *p* < 0.1, Fisher’s exact test).

Intestinal commensals have adapted to the environment and developed complex ecological networks with other bacteria to acquire nutrients. Bacteria have to compete for nutrients in the gut, especially strains which belong to the same species and require similar nutrients. In this study, 63 cultivated isolates were screened for their biochemical features in order to investigate their metabolic interactions in the gut (Figure 5, Supplementary Materials Table S7).

Consistent with the biochemical assay, *E. coli* strains exhibited considerable variability in the utilization of diverse carbohydrates. All tested strains were capable of fermenting trehalose and hydrolyzing β-galactosides into monosaccharides. Most of the strains were lactose-positive (*n* = 58) and could utilize glucuronides (*n* = 58), melibiose (*n* = 59), as well as sugar alcohols such as mannitol (*n* = 62) and D-sorbitol (*n* = 58). According to our previous data on CFU counting [40], lactose-negative strains comprised a lower proportion (7.8 ± 9.3%) of *E. coli* compared to lactose-positive strains (53.1 ± 38.2%) (Mann–Whitney U test, *p* ≤ 0.01). At the same time, strains utilizing D-adonitol (*n* = 9) and D-arabitol (*n* = 9) were predominant (71.2 ± 37.5% vs. 45.9 ± 38%) within the *E. coli* community in human feces (Mann–Whitney U test, *p* < 0.1). Therefore, successful colonization with a dense and diverse intestinal microbiota is highly dependent on the ability of bacteria to compete for nutrients. Less than half of *E. coli* fermented raffinose (*n* = 25) and sucrose (*n* = 20), while eight strains utilized dulcitol and two bacteria could digest cellobiose. Salicin was fermented by only two CD-derived strains. Aesculin-hydrolysing *E. coli* accounted for 11% of all tested strains (*n* = 7). Only four strains (2 CD-derived and two from the controls) could use citrate on Simmons’ agar. With the exception of two healthy-derived strains, lysine was decarboxylated in all *E. coli*, whereas arginine and ornithine were decarboxylated in less than half of the strains (*n* = 39 and *n* = 19, respectively). Interestingly, hydrogen sulfide production was found to be associated with CD strains (*n* = 3, *p* < 0.1). The metabolic features of the strains were not associated with the disease location in patients. Such a variety of metabolic capabilities of bacteria may be associated with high inter- and interspecies competition for nutrients, contributing to intestinal colonization and resistance to elimination.

## 4. Discussion

In this study, we comprehensively typed 63 cultivated *E. coli* from CD patients with different disease locations and healthy volunteers, revealing high genetic and phenotypic heterogeneity in the strains. We found up to five *E. coli* strains per individual human host, with one predominant strain, which is consistent with previous studies into the diversity of the fecal *E. coli* [67]. To expand the understanding of strain-level diversity, we applied computational analyses of shotgun metagenome data and assembled 18 metagenome-associated *E. coli* genomes. The phylogenetic distribution within both groups was characterized by a majority of A and D phylogroups, followed by B1 phylogroup strains, which is inconsistent with previous reports, where A and B2 groups dominated in human and animal feces [67,68]. However, it has been shown that the proportion of B2 phylogroup strains differs between populations and may be due to geographic conditions, dietary factors or host genetic factors [69,70]. It is suggested that strains of the A, B1, and D phylogenetic groups are mostly commensal and have to acquire virulence factors to become pathogenic, while the strains of phylogroup B2 are potentially more virulent [71,72] and more frequent in IBD patients [73]. However, a previous report has shown that *E. coli* from patients with the inflamed and normal ileum were similarly distributed among phylogroups [74], which was confirmed in the current study. Further MLST analysis discovered the most common genotypes, ST10 and ST69, among the identified *E. coli* present in both cohorts. These sequence types are commonly associated with humans and food animals [75,76], and serve as intestinal colonizers, causing human infections [77,78,79]. Indeed, according to our data, strains of ST10 and ST69 genotypes harbored several virulence genes more frequently than other sequence types but were not CD-associated.

The serotype-based method illustrates the limitations in the identification of pathogenic strains associated with CD. A wide diversity of serotypes was observed in both of the studied cohorts, but none of the specific serotypes were associated with CD patients. The results of virulence gene analysis failed to distinguish the virulent strains associated with CD. These findings indicate that genomic content may not reflect the ability of the strains to cause the disease. This idea is supported by previous data demonstrating that the probiotic strain Nissle 1917 is genetically close to the uropathogenic *E. coli* CFT073 [80], can induce DNA double-strand breaks and chromosomal abnormalities in eukaryotic cells [81] and can cause disease under certain conditions [82]. Moreover, a recent study demonstrated that *E. coli* strains that inhabit the gut of healthy subjects possess genetic determinants of virulence similar to the potential pathogens [83]. Thus, there is a thin line between commensalism and pathogenicity. These virulence determinants seem to contribute to bacterial competitiveness and gut colonization, rather than directly induce the disease. It is possible that impaired tolerance to the commensal bacteria colonizing the host triggers shifts in pathogen recognition, leading to disease development. Indeed, we showed that different *E. coli* strains coexist in the human gut and participate in the formation of a host-specific community, but they are subject to numerous selective pressures. It is known that genetic diversity affects the ability to adapt to novel ecological conditions [84]. The ability to compete with neighbors and resist various chemicals and antibiotics is crucial for survival. In this study, bacteriocin-producing systems, conferring an advantage in terms of dominance, were observed in genomes of 26 strains. Almost half of these strains exhibited phenotypic antagonistic activity against the target strain and obtained better results than other *E. coli* in their hosts.

Along with bacteriocin production, the metabolic flexibility discovered in *E. coli* also promotes bacterial survival and intestinal colonization, but may occasionally lead to intestinal disorders. According to previous reports [39,85,86], an increased proportion of sulfate-reducing bacteria affects IBD through the overproduction of H_2_S, causing mucus degradation and intestinal inflammation. It is assumed that hydrogen sulfide contributes to bacterial survival in the intestine, increasing their resistance to antibiotics, and protecting against reactive oxygen species and immune-mediated killing [87,88,89]. However, at elevated concentrations, hydrogen sulfide may become toxic to the host. Although all strains possessed genes for H_2_S production, only three strains from one CD patient were positive in a biochemical assay, so can we speculate that these H_2_S-producing *E. coli* may contribute to an increase in total hydrogen sulfide in the gut. However, it has not yet been established whether a specific *E. coli* strain utilizes the same nutrients and produces gases when it grows in single culture and when it coexists with other commensal bacteria colonizing the IBD intestine.

In the current study, we observed the metabolic differences promoting fecal bacteria to successfully compete at the individual strain level. Though the fecal microbiota differs from the bacterial mucosal community, which is more directly involved in the CD pathogenesis, it may contribute to disease-associated changes in the host. An imbalance in the proportion of coexisting strains and changes in their interactions with each other and other commensals may affect hosts and disrupt intestinal homeostasis.

The main limitation of the study is that the isolated *E. coli* strains have a fecal origin, rather than mucous. It would be interesting to expand the collection with more samples as well as biopsies to better understand the complexity of the intestinal community. In addition, a closer look at the host–bacteria interaction may shed light on the CD pathogenesis. Taking these limitations into account, further investigations into the physiological responses of bacteria to the host are required.

## 5. Conclusions

This study contributes to a better understanding of the bacterial interactions in the human gut, which is crucial for revealing their role in the pathogenesis of Crohn’s disease. Whole-genome sequencing, along with metagenome-associated *E. coli* genome reconstruction, allows for a comprehensive characterization of the intestinal community composition and strain-level diversity, demonstrating the remarkable intra- and intraindividual genetic diversity of *E. coli*. We showed that different *E. coli* strains coexisting in the human gut of CD patients and healthy individuals exhibit similarities regarding the distribution of virulence determinants, antibiotic resistance genes, and bacteriocin-producing systems. We revealed a high metabolic flexibility in the strains, which promotes their survival and gut colonization. These observations suggest that bacterial efforts to successfully proliferate and outcompete other bacteria under intestinal conditions may implicitly influence hosts by reducing their tolerance for asymptomatic bacterial colonization and leading to disease development.

## Figures and Tables

**Table 1:** Patient metadata.

Groups	CD Patients	Healthy Controls
Total number of subjects	40	42
Age (years)	19–61	20–65
Gender		
Female	22	23
Male	18	19
Location		
Ileal	4	NA
Colonic	22	NA
Ileocolonic	14	NA
Behaviour		
Non-stricturing, non-penetrating	21	NA
Stricturing	8	NA
Penetrating	3	NA
Stricturing, penetrating	4	NA
Non-penetrating, stricturing	4	NA

NA: not applicable.

## Data Availability

Sixty-three whole-genome sequences and 18 metagenome-assembled *E. coli* genomes described in this paper were deposited at GenBank under BioProject accession number PRJNA560176.

## Supplementary Materials

The following are available online at https://www.mdpi.com/article/10.3390/microorganisms9061299/s1, Table S1: Typing of *E. coli* from CD patients and healthy individuals according to different methods. Table S2: Prevalence of virulence-associated genes in cultivated *E. coli* strains from CD patients and healthy individuals. Table S3: Prevalence of virulence-associated genes according to the most common sequence types (ST10, ST69) by Achtman’s scheme in all cultivated *E. coli* strains from CD. Table S4: Distribution of bacteriocins encoding genes identified in 28 *E. coli* strains from CD patients and healthy individuals. Table S5: Susceptibility to different antibiotics according to disc-diffusion assay in *E. coli* strains from CD patients and healthy individuals. Table S6: Antimicrobial resistance determinants identified in *E. coli* strains from CD patients and healthy individuals. Table S7: Prevalence of biochemical features in *E. coli* strains from CD patients and healthy individuals.
